# Spatial trend, environmental and socioeconomic factors associated with malaria prevalence in Chennai

**DOI:** 10.1186/1475-2875-13-14

**Published:** 2014-01-08

**Authors:** Divya Subash Kumar, Ramachandran Andimuthu, Rupa Rajan, Mada Suresh Venkatesan

**Affiliations:** 1 Centre for Climate Change and Adaptation Research, Anna University, Chennai, Sardar Patel Road, Guindy 600 025, Chennai, Tamil Nadu, India; 2 Department of Geography, University of Madras, Chepauk 600 015, Chennai, Tamil Nadu, India

**Keywords:** Malaria, Prevalence, Hotspot analysis, Environmental, Social factors, Chennai

## Abstract

**Background:**

Urban malaria is considered to be one of the most significant infectious diseases due to varied socioeconomic problems especially in tropical countries like India. Among the south Indian cities, Chennai is endemic for malaria. The present study aimed to identify the hot spots of malaria prevalence and the relationship with other factors in Chennai during 2005-2011.

**Methods:**

Data on zone-wise and ward-wise monthly malaria positive cases were collected from the Vector Control Office, Chennai Corporation, for the year 2005 to 2011 and verified using field data. This data was used to calculate the prevalence among thousand people. Hotspot analysis for all the years in the study period was done to observe the spatial trend. Association of environmental factors like altitude, population density and climatic variables was assessed using ArcGIS 9.3 version and SPSS 11.5. Pearson’s correlation of climate parameters at 95% and 99% was considered to be the most significant. Social parameters of the highly malaria prone region were evaluated through a structured random questionnaire field survey.

**Results:**

Among the ten zones of Chennai Corporation, Basin Bridge zone showed high malaria prevalence during the study period. The ‘hotspot’ analysis of malaria prevalence showed the emergence of newer hotspots in the Adyar zone. These hotspots of high prevalence are places of moderately populated and moderately elevated areas. The prevalence of malaria in Chennai could be due to rainfall and temperature, as there is a significant correlation with monthly rainfall and one month lag of monthly mean temperature. Further it has been observed that the socioeconomic status of people in the malaria hotspot regions and unhygienic living conditions were likely to aggravate the malaria problem.

**Conclusion:**

Malaria hotspots will be the best method to use for targeting malaria control activities. Proper awareness and periodical monitoring of malaria is one of the quintessential steps to control this infectious disease. It has been argued that identifying the key environmental conditions favourable for the occurrence and spread of malaria must be integrated and documented to aid future predictions of malaria in Chennai.

## Background

According to the World malaria report 2011, there were about 216 million cases of malaria and an estimated 655,000 deaths in 2010 in 106 malaria endemic countries, including India [1]. It has been studied that the disability adjusted life-years lost due to malaria in India was 1.86 million years, and that each Indian Rupee invested by the National Malaria Control Programme equals a rich dividend of 19.7 Indian Rupees [2]. Further, the wide topological and climatic diversity and species diversity of malaria vectors makes India as a model field to examine interactions among elements of the malaria triangle: host-vector-parasite with the associated environmental parameters [3].

In India, Chennai city has become an endemic area for malaria in the past few decades. Nearly 70 percent of the malaria cases recorded in the State of Tamil Nadu occurs in Chennai City alone, the problem being more acute in north-east coastal areas [4]. *Anopheles stephensi*, the mosquito that transmits malaria in Chennai, breeds in clear water. A total of 0.28 million permanent breeding sources have been identified in Chennai in 2011, which included wells, open over-head-tanks and sumps, allowing mosquitoes to breed in and transmit malaria [4]. Malaria prevalence studies have not been carried out for Chennai city, as many researchers have studied in other places [5-7]. The aim of the study was to understand the malaria prevalence in Chennai and to delineate the possible reason behind it. A multidisciplinary approach involving geographical, statistical and sociological techniques has been employed in this study for a period of 2005 to 2011.

## Methods

### Study area

**Figure 1:**
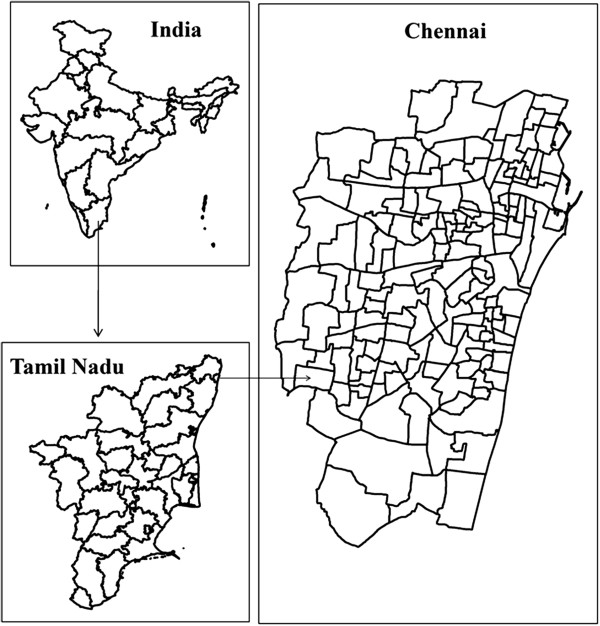
Study area.

Chennai is located on the northeast end of Tamil Nadu on the seashore, off the Bay of Bengal. It lies between 12°9' and 13°9' N latitude and 80°12' and 80°19' E longitude. Chennai district, limited by the Corporation boundary consists of ten zones with 155 wards (2011) (Figure 1). Two rivers, the Adyar and the Cooum traverse the city and drain fresh waters into the Bay of Bengal. In addition to the rivers, the Buckingham canal runs along the coast of the city. Most of the water resources have been polluted in recent years due to rapid urbanization, industrialization, increased commercialization and migration. The climate of Chennai is tropical, wet and dry. Since it lies along the coast, there is much less seasonal variation in the temperature. The average highest temperature is 33.3°C and the average lowest temperature is 23.8°C. The average annual rainfall is approximately 140 cm and the city gets most of its seasonal rainfall from the northeast monsoon winds during the mid–October to mid–December months.

### Malaria data

Data on zone wise and ward wise monthly malaria positive cases were collected from the Vector Control Office, Chennai Corporation for the year 2005 to 2011. *Plasmodium vivax* and *Plasmodium falciparum* are the predominant causative organisms. The prevalence of malaria was calculated for each ward for the spatial and temporal analysis. The term prevalence as defined in Dorland’s Medical Dictionary is the total number of cases of a disease in existence at a certain time in a designated area [8]. It is the proportion of people in the population who have malaria at a specific point in time or over a specific period of time.

### Environmental data

Detailed demographic data for the census year 2001 has been used for the study period. Altitude was estimated using a Shuttle Radar Topography Mission Digital Elevation Model (SRTM DEM) of 3 arc-second (approximately 90 m) resolution for each ward. Monthly data for all climate variables for Chennai, Nungambakkam station was obtained from the IMD (Indian Meteorological Department), Chennai.

Ward-wise spatial distributions of malaria, its relationship to elevation and population density has been assessed using ArcGIS version 9.3 software.

### Spatial and statistical analysis

Malaria hotspots have been assessed using the ArcGIS spatial statistical, mapping clusters hot spot analysis tool that calculates the Getis-Ord Gi* statistic for each ward in the dataset. The Gi* statistic for each feature in the dataset is a “*z*” score. The resultant “*z*” score is an indicator of wards with either high or low malaria prevalence clusters. Apart from this, statistical analysis of monthly malaria prevalence with monthly rainfall, monthly mean temperature and monthly mean relative humidity was conducted using the SPSS statistical package version 11.5.

### Socioeconomic survey

Structured questionnaires were developed based on the WHO guidelines on malaria indicator survey, 2005 [9]. A simple random sampling survey was conducted to examine the type of living conditions of people in the hot spots of malaria within the identified problematic zones. One hundred houses were surveyed in each of the vulnerable areas about their socioeconomic conditions, disease history and awareness on malaria prevention.

## Results

### Malaria prevalence and spatial trend

**Figure 2:**
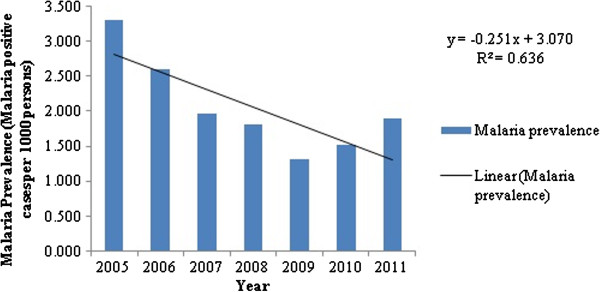
Trend of malaria prevalence in Chennai – 2005 – 2011.

**Figure 3:**
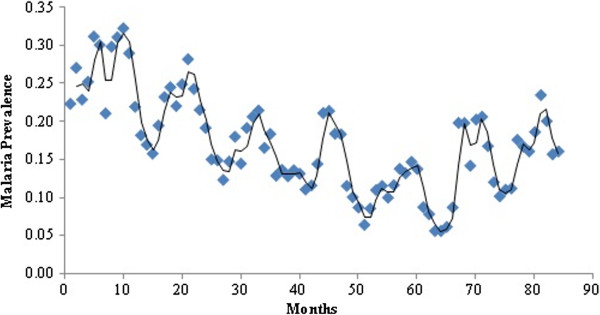
Seasonality of malaria prevalence in Chennai - 2005- 2011.

**Figure 4:**
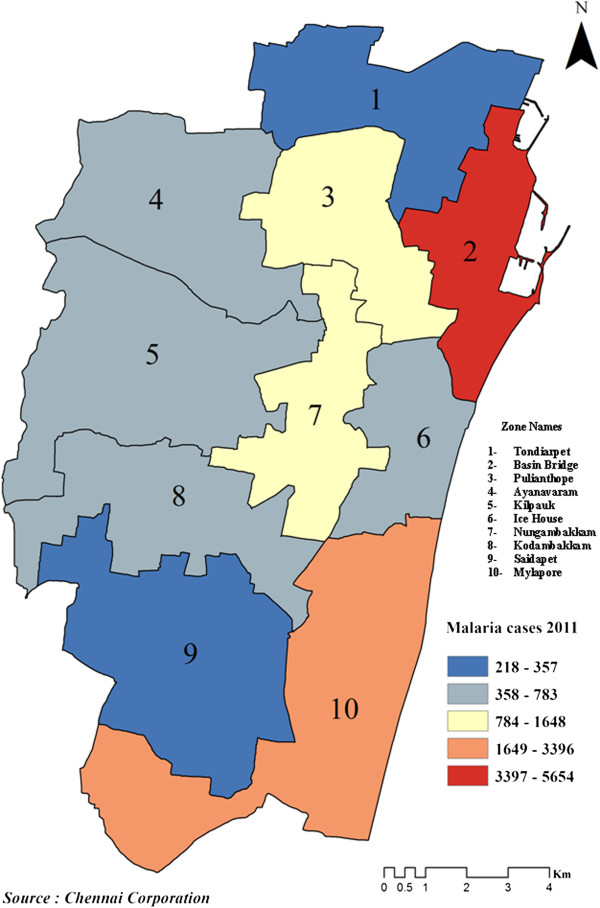
Zone wise malaria positive cases in Chennai – 2011.

**Figure 5:**
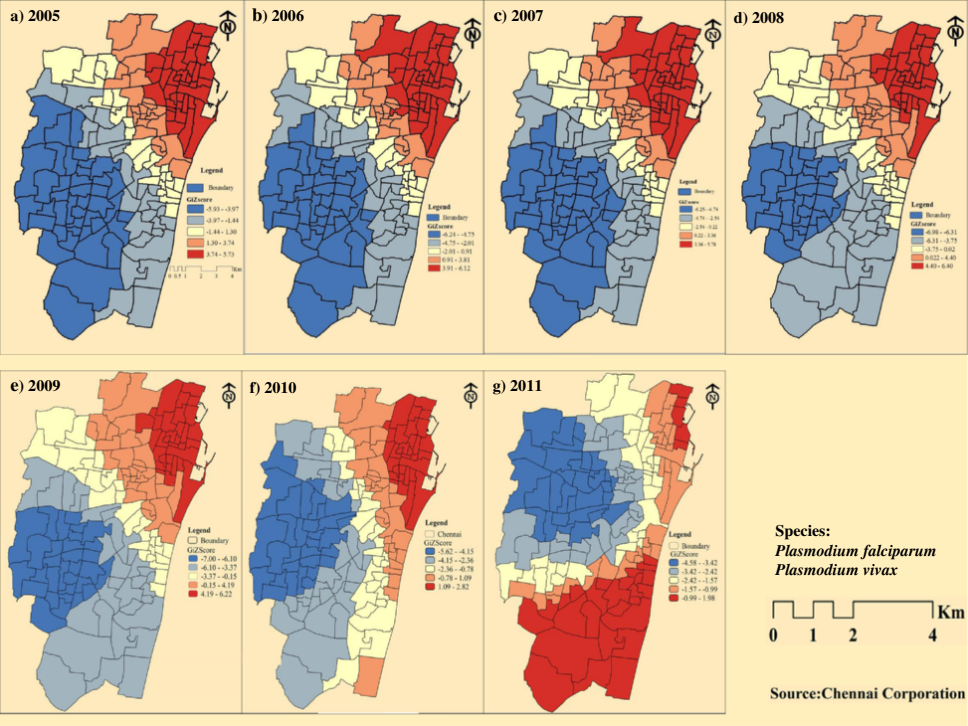
Hotspots of malaria prevalence in Chennai a) 2005, b) 2006, c) 2007, d) 2008, e) 2009, f) 2010 and g) 2011.

The temporal analysis of malaria prevalence showed that there had been a decreasing trend in the overall malaria prevalence (Figure 2), with a distinct seasonal pattern during August, September and October (Figure 3). The zone wise distribution of malaria positive cases in Chennai during the year 2011 is shown in Figure 4. It was seen that the Basin Bridge zone recorded the highest number of malaria cases followed by Adyar zone. For understanding the intricacies of malaria prevalence in Chennai, the hotspot analysis was conducted which would give an idea whether any relationship existed spatially or not. It was seen that all the wards of north eastern Chennai were malaria hotspots during 2005-2011. These hotspot wards cluster to form the Basin Bridge zone that was seen to be heavily affected by malaria. Another important observation from the hotspot analysis was that there was a sudden emergence of hot spots in southern parts of Chennai during 2011 as seen in (Figure 5a-g). A strong spatial relation of clustered occurrence to the prevalence of malaria in Chennai could be delineated which has not previously been reported.

### Association with environmental parameters

**Figure 6:**
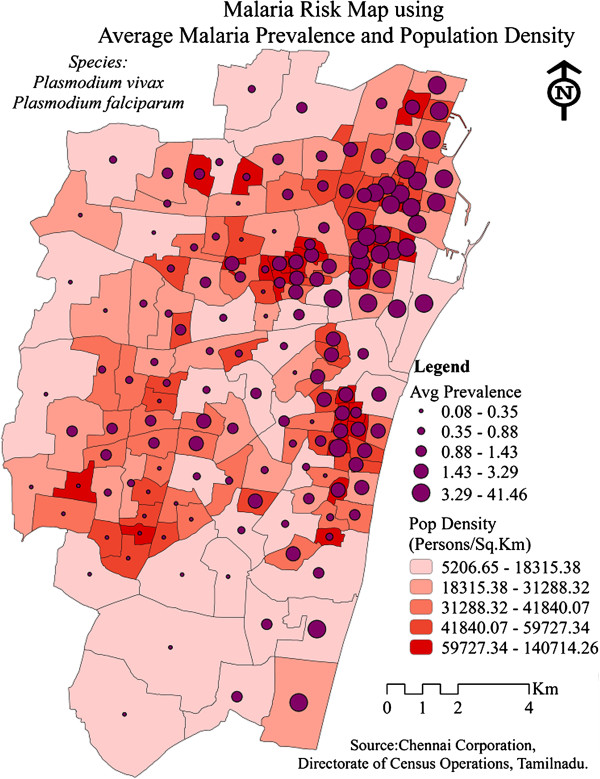
Relation of average malaria prevalence to population density in Chennai during 2005-2011.

**Figure 7:**
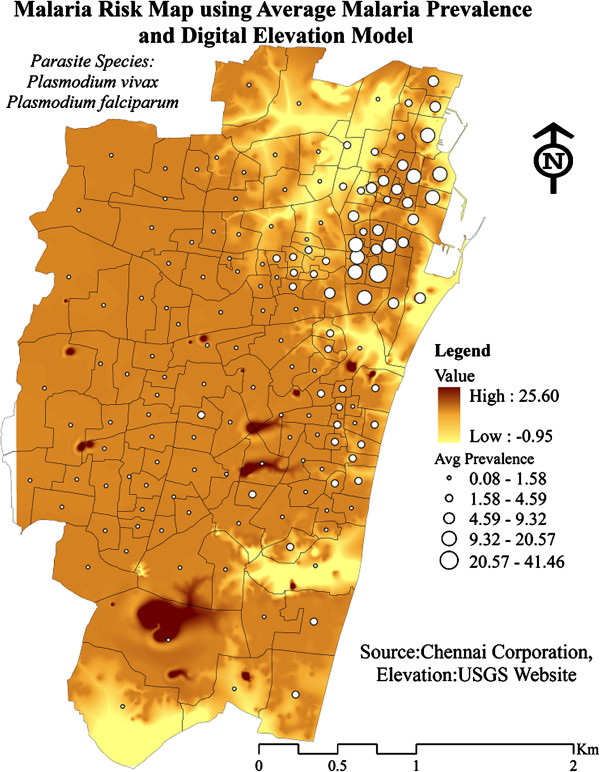
Relation of average malaria prevalence to elevation in Chennai during 2005-2011.

To further investigate this spatial pattern, population density and elevation of the wards have been considered. It was seen that densely populated and clusters of moderately populated areas showed high average prevalence of malaria during 2005-2011. These areas coincided perfectly with the malaria hot spots (Figure 6). Though there did not emerge a direct relationship between the elevation and malaria prevalence, the moderately elevated malaria prevalent regions were surrounded by low elevation areas that might have served as a reservoir for mosquito breeding (Figure 7). The role of climate variability on malaria prevalence has been described in Table 1. As depicted from the table, rainfall and one month lag of monthly mean temperature (Tmean_t-1_) had significant correlation with malaria prevalence at 99% and 95% confidence interval respectively, which shows the influence of climatic factors on vector-borne diseases.

**Table 1:** Correlation of malaria prevalence with climate parameters

		**Prevalence**	**Rain**	**Tmean**	**Tmean**_**t-1**_	**RHmean**
Prevalence	Pearson correlation	1	0.371**	0.110	0.280*	0.021
	Sig. (2-tailed)		0.001	0.321	0.010	0.853

**Correlation is significant at the 0.01 level (2-tailed).

*Correlation is significant at the 0.05 level (2-tailed).

Tmean – Monthly mean temperature.

Tmean_t-1_ – One month lag of mean temperature.

### Association with socioeconomic factors

Due to lack of resources the two most malaria prevalent zones were surveyed. It was observed through the social survey that middle and lower middle class people are the major occupants (82%) in both Adyar and Basin Bridge zones. Most of them were engaged in tertiary activities like fishing, running petty shops, renting houses and vehicular transports etc., which were not, included either under a private or public job. Most young children and elderly had suffered from malaria and were subjected to loss of effective days (>20%) of work and study. When asked to compare the usage of mosquito nets, coils and repellents as personal protection methods, majority of people preferred not to use mosquito nets (74%) which could be attributed to the perception that it is a tedious activity compared to the latter. People resorted to private medical care (72%) as they imagined that government hospitals were less equipped with facilities and less hygienic. Though most of the houses had closed overhead tanks, cleaning of the overhead tanks and sumps was done only once or twice in a year (28%). This could be a factor in higher malaria prevalence.

## Discussion

### Malaria prevalence and spatial trend

The prevalence of malaria in Chennai has been viewed through four different perspectives: the spatial, demographic, geographic and social perspectives. Various authors used different methods to find the spatial risks of many diseases and effectiveness of medical services [10-17]. A method that signifies a local hot spot of disease is the Getis Ord G* statistics. Yeshiwondim *et al*. [18] used this tool to analyse the temporal shifts in the position of hot spots in malaria in the villages of Ethiopia. It has been argued that hotspot-targeted interventions should occur at all transmission levels where resources are sufficient and rapid reductions in malaria transmission will be seen [19]. This method was employed here to examine and visualize the spatial change in significant malaria hotspots of Chennai. North Chennai had most of the hot spots during the study period and southern Chennai had recently started resulting in more hot spots. It has been observed that the high number of cases in the neighbouring wards also contributed to the hotspots.

### Association with environmental parameters

To explain the presence of numerous hotspots in North Chennai, the relationship of elevation and population density to malaria prevalence was assessed. It was marked clearly that North Chennai was a moderately elevated area but it was surrounded by low elevation areas that served as the breeding places of malarial vector. The results of other authors showed mixed association. For example Hightower *et al*. [20] demonstrated a positive association with low elevation, Reid *et al*. reported a mixed association [21] and Amek *et al*. proved that the malaria causing mosquitoes have a weak association with elevation [6]. However, Boussalis *et al*. argued that forecasting of malaria risk without land use variables probably understated the vulnerability of higher elevation communities to vector borne diseases whose range increased due to warmer temperatures [22].

Clusters of moderately populated wards were associated with higher malaria prevalence. However, these regions were places where the built up area was very dense and the vegetation was sparse [23,24]. Haque *et al*. [25] also showed that age, ethnicity, and proximity to forest, household density, and elevation were significantly and positively correlated with the malaria risk. It was further observed that the temple tanks with stagnated water and dumped garbage were spread in the entire Chennai [23]. These temple tanks were clustered in the north Chennai region with high malaria prevalence. The corporation officials claim that the presence of abandoned old buildings and inaccessible over head tanks were also one of the important reasons for the presence of hotspots in North Chennai, especially Sowcarpet and surrounding wards during the study period.

The other environmental parameters affecting malaria transmission were rainfall, temperature and relative humidity [26-31]. A significant positive correlation was found between malaria prevalence and monthly rainfall which was consistent with the study conducted by Roy *et al*. in Chennai [32], who demonstrated that the slide positive malaria cases increased with higher rainfall and subsequently decreased during the seasons with sparse rainfall during the survey period of January 2002 to December 2004. No significant relationship was seen with monthly mean temperature and monthly mean relative humidity. Some authors showed that one to three months lag temperature correlated with malaria prevalence [33,34].

Similarly there was a significant relationship with a one month lag of monthly mean temperature. This was due to the fact that there was approximately one month period in the course of the malaria infection cycle from larvae becoming an infectious mosquito to mosquito biting human and finally human developing malaria symptoms [28].

### Association with socioeconomic factors

**Figure 8:**
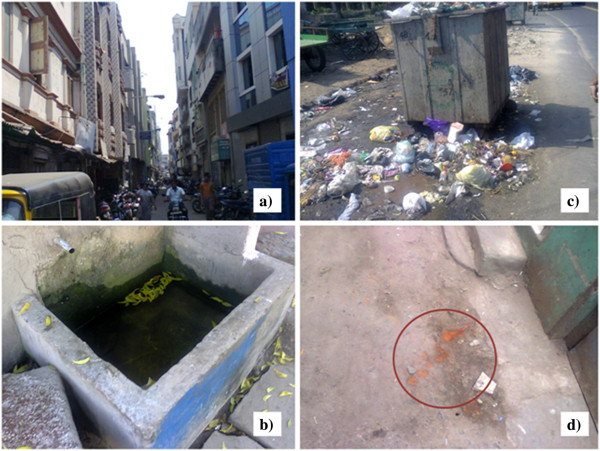
Living conditions in study area (a) Clustered buildings, (b) Practice of storing water, (c) Garbage outflow and (d) Spitting in open spaces.

Socioeconomic status of the people may also determine the extent of malaria prevalence. For instance a stronger negative association was delineated for *P. falciparum* malaria and socioeconomic status by Bui *et al*. and Levin *et al*. [35,36]. Researchers have shown that the wealth index was negatively associated with the parasitaemia risk [37,38]. The questionnaire survey showed that most of the residents are middle class people. The habit of storing drinking water was more popular and mosquito nets were less favoured. It was observed that though the houses were clean, the surrounding area was very dirty and polluted by garbage outflows (Figure 8a-d). Spitting and open defecation were also common in places of North Chennai which were hotspots of malaria.

People from the hotspot regions were mostly migrants from northern India and frequently travel to their native lands. Since this part of Chennai is a commercial hub with a tradition in export–import activities, disease spread was spontaneous and contagious. Extensive awareness among traders regarding usage of community toilets, proper disposable of waste, the avoidance of spitting and proper water storing practices are required to improve the conditions of people living in the malaria hotspot regions of Chennai. The reason for an increase in the number of hotspots even in the southern regions of Chennai may be the construction of new buildings for service sector companies and residential complexes. The wet concrete and the curing process create puddles of water that serve as breeding sites for the malaria vector.

## Conclusion

Malaria prevalence is a good indicator of the persistence of this disease in Chennai during the study period 2005-2011. From the purview of spatial extent, the hotspot analysis shows a strong spatial relationship of malaria prevalence in Chennai. Environmental factors responsible for the presence of malaria hotspots include moderately elevated areas surrounded by low elevation regions and clusters of moderately populated and densely clustered built-up areas. Prediction of malaria hotspots in Chennai could be the further scope of research in this direction. Monthly rainfall and one month lag of monthly mean temperature show significant correlation with malaria prevalence in Chennai, however the socioeconomic factors and lack of awareness on sanitation and hygiene is a more contributing factor for malaria prevalence. A comprehensive outlook on the environmental conditions favourable for the occurrence and spread of malaria along with spatial characteristics must become a part of the regular reporting and monitoring to aid future predictions of malaria in urban areas like Chennai for effective control and mitigative measures.

## Abbreviations

IMD: Indian meteorological department; SRTM DEM: Radar topography mission digital elevation model; Tmeant-1: One month lag mean temperature; WHO: World Health Organization

## Competing interests

The authors declare that they have no competing interests.

## Authors’ contribution

SKD has made substantial contributions to the design of the study, acquisition of data, analysis and interpretation of data and drafting the manuscript. AR was involved in the design of the study and instrumental in procuring the data. RR participated completely in doing the spatial analysis and interpretation of spatial data and conducting the socioeconomic surveys. VMS contributed in revising the manuscript critically for important intellectual content. All authors read and approved the final manuscript.
